# Assessment of Awareness and Practices Related to Burn Injury First Aid Among the General Public: Cross-Sectional Study in Taif, Saudi Arabia

**DOI:** 10.7759/cureus.45912

**Published:** 2023-09-25

**Authors:** Muhanna A Alhusayni, Naif M Alotaibi, Ahmed A Alshaer, Abdulelah Alnefaie, Majed M Alotaibi, Abdul Rahman R Albogami, Turkey B Juohari, Yasser Alnofaiey

**Affiliations:** 1 Department of Medicine, Taif University, Taif, SAU; 2 Department of Radiology, United Doctors Hospital, Jeddah, SAU; 3 Department of Internal Medicine, College of Medicine, Taif University, Taif, SAU

**Keywords:** saudi arabia, general public, practices, awareness, first aid, burn injury

## Abstract

Background

Burn injuries can be highly traumatic and harmful, leading to significant mortality rates, extended hospital stays, deformity, and incapacity. In the long term, they may also result in rejection, social stigma, and psychiatric issues. This study aimed to estimate the awareness and practices related to burn injury first aid among the general public in Taif, Saudi Arabia.

Methods

This is an online cross-sectional survey in Taif, Saudi Arabia. An online self-administered questionnaire was distributed to the adult population, comprising individuals aged 18 years and older, of both genders, from June 2023 to August 2023. The questionnaire consisted of 24 questions divided into demographics and first aid for burns. The Scientific Research Ethics Committee at Taif University, Taif, Saudi Arabia, obtained the ethical approval for the study.

Results

A total of 531 individuals were included in the study. About half were male (58.4%) and in the age group of 22-29 years (52%). Out of that number, 24.1% were medical field students. About one-third of the respondents had participated in a burn training course (33.7%), and 73.8% reported experiencing a burn injury before, either to themselves or their family. Most respondents chose to treat the burn area using honey, and only 15.6% knew that they should administer water to a burn injury for 10 to 15 or >15 minutes. Most of the participants reported an excellent knowledge level (62.9%). Only 8.7% had an excellent practice level. The total knowledge and practice score was significantly associated with participation in the burn training course (P-value < 0.001 and 0.015, respectively). The work nature and prior experience with a burn injury were significantly associated with the knowledge scores (P-value=0.003, for both). Monthly income and the work nature also correlated with the practice total score (P-value=0.023 and <0.001, respectively).

Conclusion

Most participants had an excellent knowledge level, however, most of them reported poor or acceptable practice scores. It highlights the need for training sessions, awareness campaigns, and dissemination of evidence-based information to bridge the gap between knowledge and practice.

## Introduction

Burn injuries can occur when hot liquids, hot solids, or flames rupture one or more layers of the skin. The term "burn" also describes the skin injury that could result from other agents such as chemical agents [1]. These injuries can have severe physical, functional, and psychological effects and are among the most significant public health problems [2, 3]. Burn injuries can result in prolonged hospital stays, deformity, incapacity, and mental disorders associated with deformity resulting from burn damage [4, 5]. Immunocompromised individuals are at a higher risk of negative consequences from burns [6].

Globally, burn injury ranks fourth among the most frequent trauma types [7]. Moreover, burns cause about 11 million injuries and 265,000 death cases yearly [8]. According to the World Health Organization, the vast majority of burn-related injuries are reported in developing countries [9]. Burn injuries in Saudi Arabia occurred at an annual rate ranging from 100 to 500 approximately per 100,000 individuals, with households being responsible for more than three-quarters of the incidents [10].

With consistent findings, numerous studies have investigated the rate and causes of burns in Saudi Arabia. Injuries with very hot liquid or steam and fire burns were the most frequent, and homes were the primary location, with children under five years being the most affected age group. Additionally, most of these studies indicated that males were more susceptible to burns [2].

The treatment cost of burns significantly burdens the healthcare system. A previous study in the USA reported that the direct cost associated with managing burn injuries among children was more than 200 million dollars [2]. Another study conducted in Germany reported that healthcare for each burn victim costs about 300,000 dollars [11]. In the United Kingdom, the yearly cost per victim was about 75,000 dollars [12]. Nonetheless, established preventive strategies and initial interventions for burn injuries have demonstrated efficacy in diminishing the morbidity and mortality rates linked to burn incidents [13, 14].

The best initial treatment for burns involves removing clothing, adding cold water for twenty minutes, seeking assistance, and applying an aseptic bandage in order to stop damage. Cold tap water should be administered within three hours of the burn injury, with a temperature between 12-18°C [15]. For many years, various topical treatments have been used for burns, such as herbs, ice, and toothpaste. However, there is no conclusive scientific proof that they are effective [2, 16, 17]. Additionally, such remedies can worsen the wound, encourage bacterial growth, and increase the post-burn consequences [16]. Studies revealed that treating some burns with ice, especially for children and older populations, can be associated with an increased risk of tissue damage and hypothermia [18, 19].

Multiple studies have highlighted a need for more awareness regarding burn first aid in both developed and developing countries [6, 18, 20]. Similarly, studies conducted in KSA have revealed a lack of awareness among the general population regarding burn prevention and first aid management [21-24]. Therefore, we aimed to assess the level of awareness and practice of burn injuries first aid in the general public in Taif City, Saudi Arabia.

## Materials and methods

Study design

This was an online cross-sectional descriptive survey conducted in Taif, Saudi Arabia, between June 2023 to August 2023.

Study populations

The study was conducted among adult residents of Taif City, Saudi Arabia, aged 18 years and older, of both genders, to examine awareness and practice levels regarding burn injury first aid.

Data collection

We used an online self-administered Arabic questionnaire that was adapted from a similar prior study [1]. The questionnaire was distributed to the general public of Taif City, Saudi Arabia, as a link to a Google Form using social media platforms. The questionnaire included a total of 24 questions collecting data on demographic characteristics of the responders (11 questions) as well as their knowledge (seven questions) and practices (six questions) towards burn injuries first aid. Knowledge and practice-related questions were multiple-choice questions with only one correct answer for each question. Each correct answer was given 1 point, and incorrect answers were given 0 points. The total score for knowledge ranged from 0 to 7, and the total score for practice ranged from 0 to 6. A higher score indicated higher knowledge or practice levels for first aid of burns.

Statistical analysis

Data were extracted from the Google Form in Excel format. Statistical analysis of the data was conducted using the Statistical Package for Social Sciences (SPSS) software, version 29.0 (IBM Corp., Armonk, NY, USA). Descriptive statistics were used to present the data, where categorical data were described as frequencies and percentages, and numerical variables were presented as median and inter-quartile range (IQR). The factors affecting knowledge and practice scores were examined using non-parametric Kruskal-Wallis test. P-values <0.05 were considered statistically significant.

Ethical consideration

Before conducting any study-related procedures, we obtained ethical approval from the Scientific Research and Ethics Committee of Taif University, Taif, Saudi Arabia, with reference number 44-379. All participants agreed to participate in the study after being informed of its objectives. The confidentiality of collected data was maintained throughout and after the study duration.

## Results

A total of 531 individuals participated in the study. About half of the respondents were male (58.4%) and in the age group of 22-29 years (52%). Most of them were Saudi nationals (89.6%) and university-educated (73.3%). The majority of the respondents were students (29%) or medical field students (24.1%) and had a monthly income of less than 10,000 SAR (67%). Most respondents were single (74.2%) and had children under 18 years living at home (77.4%). About one-third of the respondents had participated in a burn training course (33.7%), and half of the respondents had a first-degree relative in the medical field (50.8%). Furthermore, 73.8% of the respondents reported experiencing a burn injury before, either to themselves or their family. All details are described in Table 1.

**Table 1:** Baseline characteristics of the respondents.

Characteristics	N=531	Percentage
Gender		
Female	221	41.6
Male	310	58.4
Age		
19-21	140	26.4
22-29	276	52
30-39	27	5.1
40-50	70	13.2
>50	18	3.4
Nationality		
Non-Saudi	55	10.4
Saudi	476	89.6
Educational level		
Primary	4	0.8
Intermediate	15	2.8
High school	123	23.2
University	389	73.3
Employment		
Student, other specialities	154	29
Medical Field Student	128	24.1
Government sector employee	86	16.2
Privet sector employee	47	8.9
Self-employed	22	4.1
Unemployed	94	17.7
Monthly income		
<10,000 SAR	356	67
10,000-20,000 SAR	121	22.8
21,000-30,000 SAR	34	6.4
>30,000 SAR	20	3.8
Marital status		
Single	394	74.2
Married	133	25
Divorced	3	0.6
Widowed	1	0.2
With children/adolescents/teenagers (under 18 years) living at home		
Yes	411	77.4
No	120	22.6
Participated in the burn training course		
Yes	179	33.7
No	352	66.3
Do you have a first-degree relative in the medical field?		
Yes	270	50.8
No	261	49.2
Have you ever experienced a burn injury before to yourself or your family?		
Yes	392	73.8
No	139	26.2

The knowledge and practices of participants regarding burn first aid are presented in Table 2.

**Table 2:** Knowledge and practices of respondents on burn first aid.

Knowledge statement	Correct answer	True N (%)	False N (%)
1. Burn can lead to permanent injuries.	Agree	452 (85.1)	79 (14.9)
2. Burn injuries can lead to mental disorders.	Agree	465 (87.6)	66 (12.4)
3. Covering the burned area before heading to the hospital can decrease the risk of infection.	Agree	341 (64.2)	190 (35.8)
4. Picking blisters is incorrect.	Agree	305 (57.4)	226 (42.6)
5. Burn first-aid at home can lead to a better outcome.	Agree	460 (86.6)	71 (13.4)
6. In case of an electrical burn injury, I should not touch the injured person if he/she is still in contact with an electrical current.	Agree	475 (89.5)	56 (10.5)
7. In case of an electrical burn injury, the first action is to turn off the source of electricity if possible.	Agree	475 (89.5)	56 (10.5)
Practice Statement	Correct answer	True N (%)	False N (%)
1. Washing the burned area with room temperature water is the first correct step in case of burn injuries.	Agree	262 (49.3)	269 (50.7)
2. In case of burn injury, which one of the following traditional medications will you consider applying? (Oil, Aloe, Honey, Coffee, Toothpaste, Nothing)	None	204 (38.4)	327 (61.6)
3. How to extinguish a pot of oil caught on fire. (Cover with clothes, Look for help, Leave the place immediately, Pour water on it, I don't know)	Cover with clothes	420 (79.1)	111 (20.9)
4. In case of burn injury, apply water for: (<5 minutes, 5-10 minutes, 10-15 minutes, >15 minutes)	10-15 minutes, >15 minutes	83 (15.6)	448 (84.4)
5. In case of burn injury, if your clothes were caught in the fire, you should roll on the ground.	Agree	425 (80)	106 (20)
6. In case of burn injury, it is beneficial to use antibiotics in its management.	Disagree	135 (25.4)	396 (74.6)

Only 64.2% were aware that concealing the injured region before proceeding to the medical facility might lower the potential for infection in the event of a burn occurrence in case of burn injury. Additionally, approximately half (57.4%) knew picking blisters is incorrect in burn cases. On the other side, almost all the participants knew that burns could lead to permanent injuries or mental disorders.

**Figure 1:**
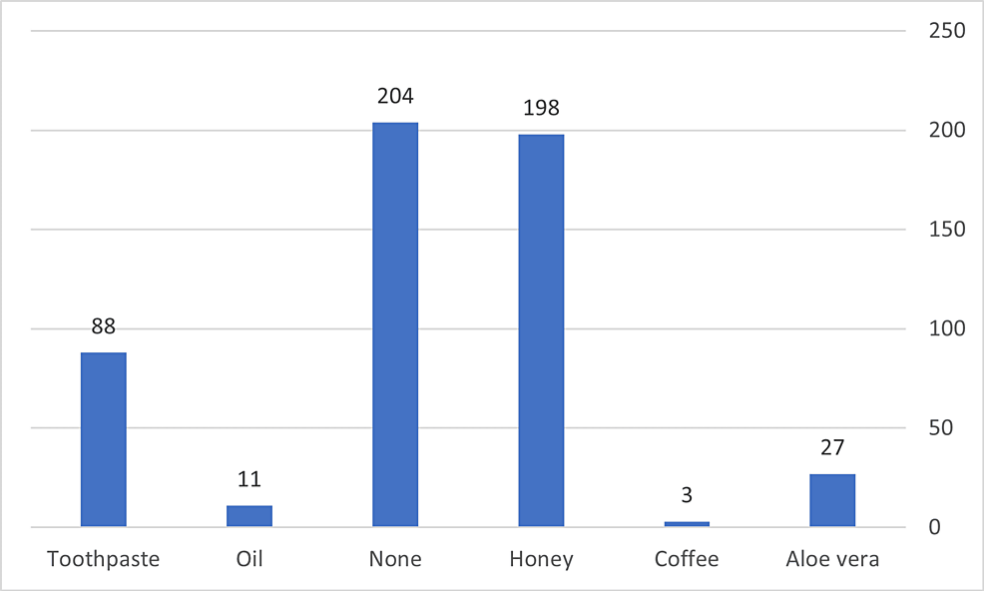
Folk medicines used for burn injuries.

**Figure 2:**
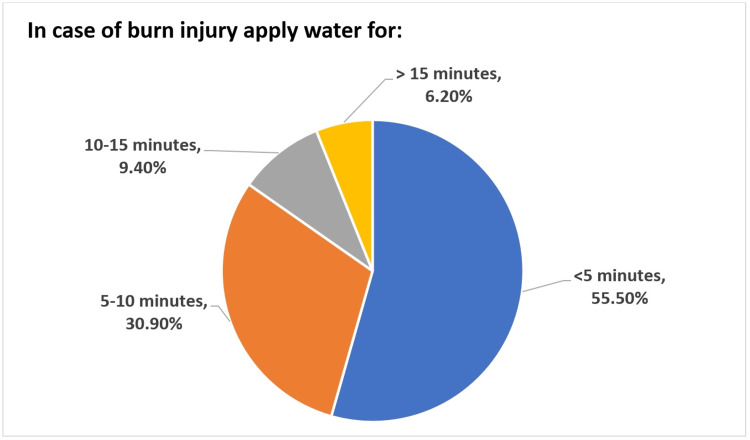
Number of minutes that the participants would apply water to the burned area.

Most respondents chose to treat the burn area using honey (198), followed by toothpaste (88). However, about one-third of them did not favor the treatment of burns with traditional remedies (204, 38.4%) (Figure 1). Moreover, only 83 (15.6%) were aware that it is important to apply water to a burn injury for 10 to 15 minutes or more (Figure 2). A high proportion (74.6%) thought using antibiotics to manage burns was beneficial.

**Figure 3:**
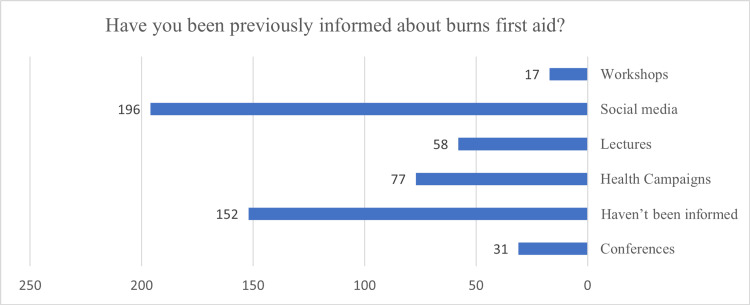
The reported source of information about burn injuries first aid.

In terms of the participants' source of information regarding burns first aid (Figure 3), social media (196), followed by health campaigns (77) and lectures (58), were the most common sources of information reported by the participants. On the other hand, a high proportion of them (152) reported that they were not informed about the burns first aid.

Based on the results (Table 3), the majority of the participants, 334 (62.9%), had an excellent knowledge level. In addition, most of them (74.2%) had poor and acceptable practice scores.

**Table 3:** Prevalence of knowledge and practices related to burn first aid.

Statement	N (%)
Knowledge score: Median (IQR)	6 (5.7)
Level of knowledge	
Poor	47 (8.9)
Acceptable	51 (9.6)
Good	99 (18.6)
Excellent	334 (62.9)
Practice score: Median (IQR)	3 (2.4)
Level of practices	
Poor	197 (37.1)
Acceptable	197 (37.1)
Good	91 (17.1)
Excellent	46 (8.7)

Several variables were associated with the total knowledge score of the participants. Participation in the burn training course was a significant factor that impacted the knowledge level among participants. Particularly, the mean rank of knowledge score for the subjects who participated in the burn training course (305) was significantly higher than the mean rank of those who did not participate (246) (P-value<0.001).

Likewise, the work nature and the prior experience with a burn injury were associated with the knowledge level among participants (P-value=0.003, for both). Self-employed participants reported a less knowledge median than others. Those who have experienced a burn injury reported a higher mean rank of knowledge (277) than those who have not experienced a burn injury before (234). Full details are illustrated in Table 4.

**Table 4:** Factors affecting participants’ level of knowledge about burn injuries.

Characteristics	Knowledge score (7) Median (IQR)	P-value
Gender		
Female	6 (5.7)	0.288
Male	6 (5.7)	
Age		
19-21	6 (5.7)	0.147
22-29	6 (5.7)	
30-39	5 (5.6)	
40-50	6 (5.6)	
>50	6 (5.7)	
Nationality		
Non-Saudi	6 (5.6)	0.138
Saudi	6 (5.7)	
Educational level		
Primary	6.5 (5.7)	0.654
Intermediate	6 (2.7)	
High school	6 (5.7)	
University	6 (5.7)	
Employment		
Student	6 (5.7)	0.03
Medical Field Student	6 (5.7)	
Government sector employee	6 (5.7)	
Privet sector employee	6 (5.6)	
Self-employed	5 (4.7)	
Unemployed	6 (5.7)	
Monthly income		
<10,000 SAR	6 (5.7)	0.523
10,000-20,000 SAR	6 (5.6)	
21,000-30,000 SAR	6 (5.7)	
>30,000 SAR	6 (5.7)	
With children/adolescents/teenagers (under 18 years) living at home		
Yes	6 (5.7)	0.874
No	6 (5.7)	
Participated in the burn training course		
Yes	6 (5.7)	<0.001
No	6 (5.6)	
Do you have a first-degree relative in the medical field?		
Yes	6 (5.7)	0.51
No	6 (5.7)	
Have you ever experienced a burn injury before to yourself or your family?		
Yes	6 (5.7)	0.003
No	6 (5.6)	

As shown in Table 5, the practice median among Saudi participants was significantly higher than other nationalities (3 vs. 2, P-value < 0.001). Participants' work nature was significantly correlated with the total practice score; self-employed individuals reported less median score (P-value <0.001). Other factors also were associated with the total score of the practice among participants, including monthly income and whether they had participated in the burn training course (P-value=0.023 and 0.015, respectively). The mean rank of practice scores among individuals with a monthly income of 21,000-30,000 SAR was higher than others. The mean rank of the practice score among subjects who participated in the burn training course (288) was higher than the mean rank of those who did not participate (255).

**Table 5:** Factors affecting participants’ level of practice regarding burn injuries.

Characteristics	Practices score (6) Median (IQR)	P-value
Gender		
Female	3 (2.3)	0.659
Male	3 (2.4)	
Age		
19-21	3 (2.4)	0.296
22-29	3 (2.3)	
30-39	3 (2.3)	
40-50	3 (2.4)	
>50	3 (3.4)	
Nationality		
Non-Saudi	2 (2.3)	<0.001
Saudi	3 (2.4)	
Educational level		
Primary	2 (2.4)	0.208
Intermediate	2 (2.3)	
High school	3 (2.4)	
University	3 (2.4)	
Employment		
Student	3 (2.3)	<0.001
Medical Field Student	3 (3.4)	
Government sector employee	3 (2.4)	
Privet sector employee	3 (2.4)	
Self-employed	2 (2.3)	
Unemployed	2.5 (2.3)	
Monthly income		
<10,000 SAR	3 (2.3)	0.023
10,000-20,000 SAR	3 (2.4)	
21,000-30,000 SAR	3 (3.4)	
>30,000 SAR	3 (2.4)	
With children/adolescents/teenagers (under 18 years) living at home		
Yes	3 (2.4)	0.83
No	3 (2.4)	
Participated in the burn training course		
Yes	3 (2.4)	0.015
No	3 (2.3)	
Do you have a first-degree relative in the medical field?		
Yes	3 (2.3)	0.351
No	3 (2.4)	
Have you ever experienced a burn injury before to yourself or your family?		
Yes	3 (2.4)	0.202
No	3 (2.4)	

## Discussion

Early management of burn injuries is crucial for the survival of burn victims. Transferring burn cases to definitive care facilities could take hours to days. Therefore, adequate first aid and urgent management could significantly minimize the consequences and enhance the survival rate [25-27]. The present study aimed to explore the awareness level and practice of burn injury first aid among the general population in Saudi Arabia to examine if the general public requires first aid awareness programs.

A Saudi study reported that 52.7% of their participants had an excellent level of knowledge, but only 1.9% had an excellent practice level [28]. Furthermore, Wallace et al. [29] indicated that 50% of participants had sufficient knowledge regarding burns first aid. In the present study, most participants (62.9%) had an excellent knowledge level. This may be because 24.1% of the individuals were medical students. However, this proportion was not translated into a remarkable practice level, and only 8.7% of them had an excellent practice level. In addition, only 17.1% reported good practice scores. This highlights the need to conduct awareness campaigns to address adequate first-aid and burn avoidance measures in Saudi Arabia, with a special focus on practice-oriented campaigns.

Ice, oil, herbal medicines, honey, vinegar, toothpaste, flour, and eggs are among the most commonly traditional materials utilized in burns management [2,15]. Particularly, it was revealed that toothpaste and honey are highly applied as a home remedy [4]. This traditional practice could be harmful because it may lead to worsening pain, tenderness, skin sloughing, and high susceptibility to infection [1]. Participants of previous studies recommended the application of toothpaste, egg, honey, or tomato paste in burns treatment [4,21]. Moreover, more than 25% of participants reported honey as the first choice of traditional remedies in burns treatment in another study [28]. Another study conducted in Saudi Arabia revealed that about 70% of the participants treated use honey alone in case of burns [2]. In other countries like the UK, it also found that 4% used toothpaste for burns [30]. In a similar manner, our findings revealed that about two-thirds (61.6%) of the participants recommended traditional remedies for treating burns. Most participants chose to treat the burn area using honey, followed by toothpaste.

Only 15.6% of the participants in our study were aware of the requirement to apply water to burns for a duration of 10 to 15 minutes or more. Another study yielded comparable findings, indicating that about 12.5% of the study participants were aware of the necessity of irrigating a burn injury for 10 to 15 minutes or more [28]. Similar findings were observed in recent studies conducted in Saudi Arabia and Cambodia, with awareness rates of only 5.8% and 13% among the respective study subjects concerning the need for water application to burn areas [2,6].

A previous study by Awan et al. [28] revealed that 78.8% of the participants agreed that antibiotics could help treat burns. The study by Mortada et al. [1] reported poor knowledge of antibiotic use in case of burn injury where 82% of the responders believe that antibiotics should be used in burn injuries. In the current study, a high percentage (74.6%) thought using antibiotics to manage burns was beneficial and needed to understand antibiotics used in burn injuries. This could contribute to antibiotic resistance as the public tends to use antibiotics out of indication.

Like previous studies in Saudi Arabia, social media was the most frequently reported information source among our participants, while workshops and conferences were the least frequently reported sources [7,21,28]. This may result from the ease of accessibility of social media platforms among people of different ages. This highlights the importance of utilizing social media as a vital platform for enhancing public awareness about burn injury first aid. Furthermore, many subjects needed to be informed about burns first aid, emphasizing the importance of establishing awareness campaigns.

Interestingly, participation in the burn training course significantly affected participants' knowledge and practice levels. Similarly, a prior Saudi study found that participants who received training courses on burn first aid showed better practice scores [28]. Unlike previous research, the current study did not reveal a significant correlation between participants’ educational level and adequate burn first-aid [2]. This could be because the vast majority of our study population received high school education or held a university degree. Moreover, it was indicated in a prior study that individuals with a high monthly income got a high practice score (P-value<0.001) [28]. In the present study, monthly income was found to significantly affect the practice total score. In addition, significantly lower knowledge and practice scores were reported among self-employed individuals. This can be attributed to the lack of formal first aid training, busy schedules, and limited access to workplace safety resources, which are typically available to employees in larger organizations.

These findings reveal broad gaps in understanding practice principles among participants.

## Conclusions

The present study underscores the necessity of implementing a first aid program for burn injuries in Saudi Arabia. The majority of participants had an excellent knowledge level. However, most of them reported poor or acceptable practice scores. It highlights the need for training sessions, awareness campaigns, and the dissemination of evidence-based information to bridge the gap between knowledge and practice. By promoting appropriate first aid practices, the general population can contribute to better outcomes for burn injury cases and reduce the risk of complications.
